# Novel Bovine Serum Albumin Protein Backbone Reassembly Study: Strongly Twisted β-Sheet Structure Promotion upon Interaction with GO-PAMAM

**DOI:** 10.3390/polym12112603

**Published:** 2020-11-05

**Authors:** Andra Mihaela Onaș, Iuliana Elena Bîru, Sorina Alexandra Gârea, Horia Iovu

**Affiliations:** 1 Advanced Polymer Materials Group, University Politehnica of Bucharest, 011061 Bucharest, Romania; andra_mihaela.onas@upb.ro (A.M.O.); iuliana.biru@upb.ro (I.E.B.); garea_alexandra@yahoo.co.uk (S.A.G.); 2 Academy of Romanian Scientists, 050094 Bucharest, Romania

**Keywords:** graphene oxide (GO), polyamidoamine (PAMAM) dendrimer, bovine serum albumin (BSA), protein interaction, circular dichroism spectroscopy, secondary structure evaluation

## Abstract

This study investigates the formation of a graphene oxide-polyamidoamine dendrimer complex (GO-PAMAM) and its association and interaction with bovine serum albumin (BSA). Fourier-transform infrared spectrometry and X-ray photoelectron spectrometry indicated the formation of covalent linkage between the GO surface and PAMAM with 7.22% nitrogen content in the GO-PAMAM sample, and various interactions between BSA and GO-PAMAM, including π-π* interactions at 291.5 eV for the binding energy value. Thermogravimetric analysis highlighted the increasing thermal stability throughout the modification process, from 151 to 192 °C for the 10% weight loss temperature. Raman spectrometry and X-ray diffraction analysis were used in order to examine the complexes’ assembly, showing a prominent (0 0 2) lattice in GO-PAMAM. Dynamic light scattering tests proved the formation of stable graphenic and graphenic-protein aggregates. The secondary structure rearrangement of BSA after interaction with GO-PAMAM was investigated using circular dichroism spectroscopy. We have observed a shift from 10.9% β-sheet composition in native BSA to 64.9% β-sheet composition after the interaction with GO-PAMAM. This interaction promoted the rearrangement of the protein backbone, leading to strongly twisted β-sheet secondary structure architecture.

## 1. Introduction

Graphenic materials exhibit interesting structural features, such as exceptional mechanical, electrical and thermal properties [1], accessible chemical modification and increased specific area [2]. Due to this unique combination of properties, researchers have focused on developing novel biomedical and non-biomedical applications starting from graphene-based materials. The addition of graphene has been demonstrated to enhance certain properties of nanocomposites, such as thermal stability, electrochemical activity or mechanical strength and electrical conductivity [3]. Non-biomedical applications for graphenic materials include anodes for Li-ion batteries [4,5], gas sensors [6] or electrochemical supercapacitors [7]. Apart from those, biomedical applications have been developed, such as biosensor devices with high specificity [8,9], scaffolds for cellular growth [10] or tissue engineering [11]. Another interesting biomedical application of graphenic materials is the development of bioactive cements with improved mechanical properties for dental applications [12].

Bovine serum albumin (BSA) is a blood protein that has been intensively studied for biological and biomedical applications, due to its low price, biocompatibility and high ligand-binding properties [13]. Numerous studies have been elaborated regarding the interaction of albumins with graphenic materials, with focus on graphene oxide (GO) [14,15,16,17,18,19,20,21], aiming to elucidate their interaction mechanism. Considering the possible denaturation effect of GO on proteins, such studies are fundamental for understanding the GO influence on the structure and activity of biomacromolecules. Kuchlyan et al. highlighted the critical role of π-π stacking and hydrophobic interactions between the protein and the graphenic material with regards to protein adsorption experiments [16]. Furthermore, other researchers have focused on the pH and temperature effect of various types of albumins’ binding capacities to GO, demonstrating that the secondary structure of BSA consists majorly in α-helices after the interaction with the graphenic material [15]. However, the chemical modification of the GO surface is of the utmost importance as it may lead to different results regarding the effect upon the structural characteristics of BSA.

Dendrimers are essentially hyperbranched polymers with a nanosized spherical shape and reactive surface functionalities [22]. Polyamidoamine (PAMAM) dendrimers were given substantial interest as they include a great amount of amine groups [23], thus generating biomedical applications such as drug and DNA delivery [24], polymer gels [25] and biosensing devices [22]. PAMAM dendrimers covalently bonded to a graphene surface have been obtained and they showed remarkable potential in biosensing applications [26]. Another study showed that a PAMAM dendrimer and glycyrrhetinic acid grafting on graphene oxide improved biocompatibility and targeted gene transfection [27]. Moreover, reducing cytotoxicity of graphene nanosheets through protein or bioactive peptide coating [28,29] represents an interesting direction in graphenic surface modification.

Therefore, investigating the effect of PAMAM-functionalized GO upon the BSA structure is of great interest. This study combines the three previously-mentioned elements, each of them contributing in a specific way to the formation of a PAMAM surface-modified graphene oxide complex with BSA, established through various non-covalent and covalent interactions between the modified surface of GO and the protein chains. GO in its natural state possesses a limited number of carboxylic surface groups. Therefore, in this study, the raw material was carboxylated to extend the functionalization sites for PAMAM dendrimer linkage. The PAMAM dendrimer was covalently attached to the carboxylated surface of GO via N-(3Dimethylaminopropyl)-N’-ethylcarbodiimide (EDC) coupling. Subsequently, the protein was introduced leading to the formation of a new complex structure (GO-PAMAM-BSA). Hence, a GO substrate suitable for protein adsorption was created, demonstrating BSA linkage to the expressly modified GO surface. Moreover, the physical and chemical properties of the novel protein-graphenic complex were investigated, alongside the effects upon the secondary structure of BSA. This study not only helps elucidate the potential toxicological effects of the graphenic materials, but the design of the new graphenic system is also of interest in the development of new biosensing devices [30].

## 2. Materials and Methods

Graphene oxide (GO) powder was purchased from NanoInnova (Madrid, Spain) and was used as received. Bovine Serum Albumin (BSA, product of USA) and N-(3Dimethylaminopropyl)-N’-ethylcarbodiimide hydrochloride (EDC, product of Japan), were purchased from Sigma Aldrich and have been used without any prior purification. Poly(amidoamine) (PAMAM, product of USA) dendrimer, ethylenediamine core, generation 0 was purchased as a 20 wt.% methanolic solution from Sigma Aldrich and the methanol was removed before use. Synthesis of each material was performed in distilled water. Filtrations have been carried out on PES membrane filters with 0.45 μm pore size and dialysis was performed in 12 kDa molecular weight cut-off dialysis sacks from Sigma Aldrich.

### 2.1. Procedure

#### 2.1.1. Synthesis of GO-COOH

**Scheme 1:**
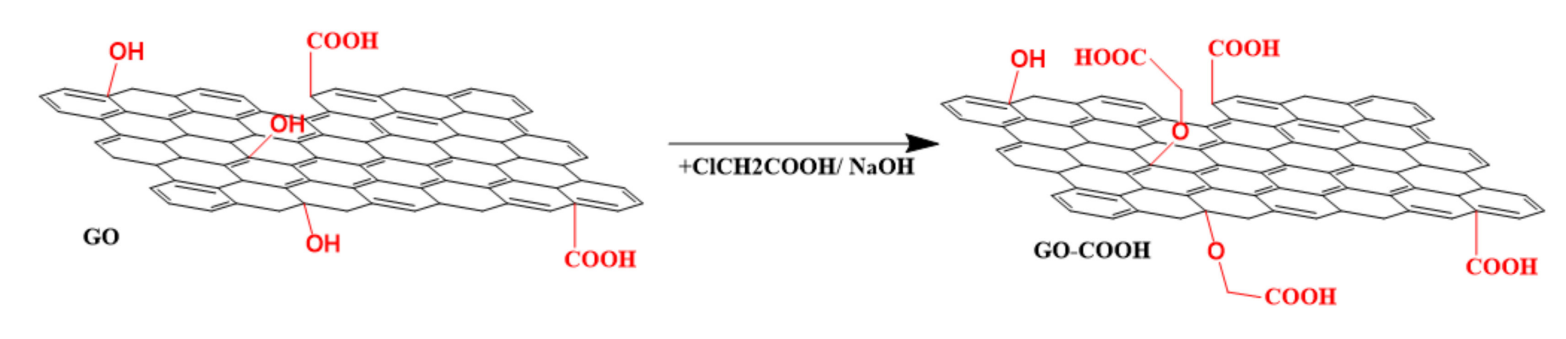
Synthesis of GO-COOH.

Carboxylated graphene oxide (GO-COOH) has been synthesized (Scheme 1) from graphene oxide by using for each 100 mg of GO 2.4 g of chloroacetic acid and 2 g of sodium hydroxide. Firstly, GO was sonicated in 150 mL of distilled water for 15 min. Sodium hydroxide and chloroacetic acid were added under continuous sonication. The mixture was kept under ultrasound dispersing treatment for 3 h, at 0–5 °C. Thereafter the mixture was neutralized, filtered, washed and dried for 72 h at room temperature [31].

#### 2.1.2. Synthesis of GO-PAMAM

**Scheme 2:**
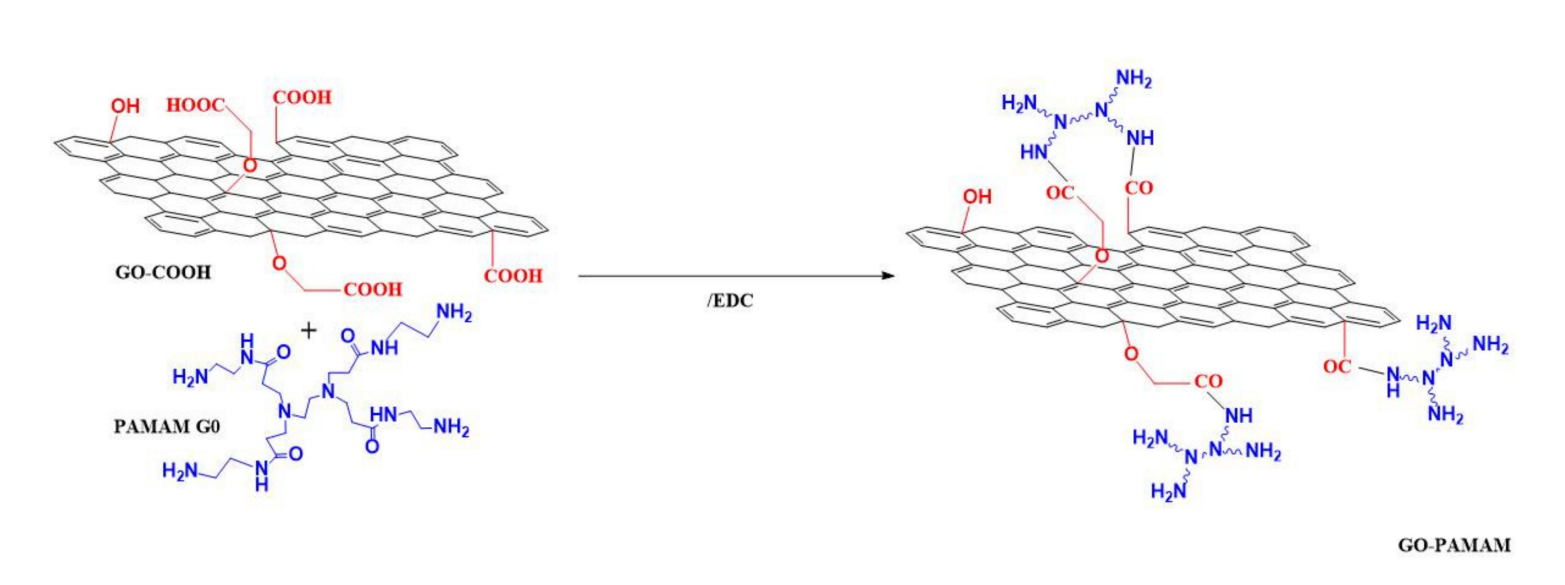
Synthesis of graphene oxide-polyamidoamine (GO-PAMAM) via EDC coupling agent, using a PAMAM dendrimer generation 0.

The covalent attachment of the PAMAM dendrimer (generation 0) to the previously purified GO-COOH was carried out via EDC coupling agent (Scheme 2). A ratio of 1:1:3 (*w/w*) between GO-COOH, PAMAM G0 and EDC was used following the next procedure: the carboxylated graphene oxide was sonicated in aqueous medium for 15 min, subsequently the coupling agent was added and after 15 min of continuous sonication the dendrimer was introduced into the mixture. To allow proper mixing, the compounds were given 24 h to react at room temperature under continuous stirring. The new graphenic material was filtered and then dialyzed for 24 h. The product was dried at room temperature for 72 h.

#### 2.1.3. Synthesis of GO-PAMAM-BSA

**Scheme 3:**
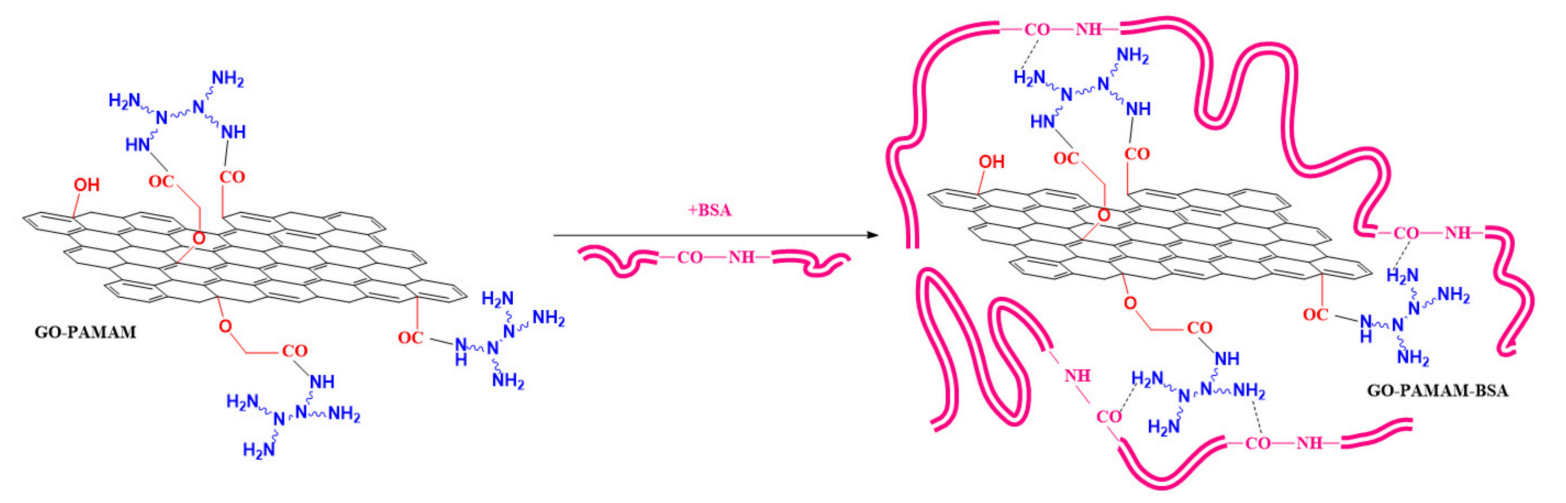
Adsorption of bovine serum albumin (BSA) protein in the GO-PAMAM complex via various interactions.

For the reaction with the protein a 1:3 (*w/w*) ratio of GO-PAMAM:BSA was used (Scheme 3). Previously synthesized GO-PAMAM was ultrasonically dispersed for 15 min, at 0–5 °C. The graphenic dispersion was allowed to reach room temperature and BSA was added. The protein was afterwards allowed to interact with GO-PAMAM for 72 h at room temperature under magnetic stirring so that the preparing conditions would not affect the native structure of BSA. The final product was freeze-dried and then washed with distilled water for the removal of the protein excess, followed by 72 h drying at room temperature.

### 2.2. Instruments

Fourier-transform infrared spectrometry (FTIR) analysis samples were prepared in a KBr pellet with a 0.5% sample concentration for each analysis. The spectral results have been collected using a Bruker Vertex 70 instrument, in the 4000–400 cm^−1^ region, using a 4 cm^−1^ resolution, accumulating 32 scans.

X-ray photoelectron spectrometry (XPS) has been carried out using a K-Alpha equipment (Thermo Scientific), with a monochromated Al Kα source (1486.6 eV), using a 2∙10^−9^ mbar pressure. In order to deconvolute the C1s XPS spectra a subtraction of the Shirley background was previously performed.

Thermogravimetric analyses (TGA) were performed using a Q500 TA instrument at a temperature range varying from 25 to 800 °C. Investigations were done using nitrogen with a 90 mL/min flow rate and a heating rate of 10 °C/min. Each sample weighed approximately 4 mg.

Raman spectral results have been recorded with the use of a Renishaw inVia Raman microscope equipment, using a 473 nm excitation laser. The laser had a 0.4 mW incident power, while using a 100x objective. All the samples had a 30 s exposure time and spectral results were registered using 3 accumulations, and a 10% laser power within the 3200–100 cm⁻^1^ range.

Circular dichroism was performed on a JASCO J-1500 circular dichroism spectrophotometer, using a 150 W Xe lamp. The data were recorded between 176–260 nm, with a 0.025 nm data pitch, 100 nm/min scanning speed and 1.00 nm bandwidth. The samples were analyzed in a 1 mm path-length quartz rectangular cell, using 3 accumulations. For the estimation of the protein secondary structure a quantitative multivariate analysis software program with a reference data set including 26 proteins was used. The mathematical method employed for the estimation was principal component regression (PCR), and the results were taken into consideration for further investigation. In this study all the CD analyses were performed using a 0.1 mg/mL protein aqueous solution.

Dynamic light scattering (DLS) was utilized to investigate the size range of the graphenic aggregates, as well as the isoelectric point of the protein and the zeta potential of the complexes. The results were determined using a Zetasizer Nano ZS (Malvern Instrument, UK). The instrument used a He−Ne laser which is linearly polarized, performing the measurements at 632.8 nm wavelength and a 173° angle for size investigations and 130° angle for zeta potential measurements. The isoelectric point of BSA was determined using the DLS technique, the measurements being performed in PBS with 1 mM NaCl addition.

The polydispersity index as well as the mean hydrodynamic diameter have been obtained by calculating the particles’ diffusion coefficient subjected to Brownian motion. Stokes−Einstein equation was employed in order to calculate those parameters:

(1) $$D_{H}\;=\frac{K_{B}T}{3\pi \eta D}$$

where: K_B_—Boltzman constant, J∙K⁻^1^, T—absolute temperature, K, η—solvent viscosity, Pa∙s

Precise measurements were performed using ultrapure water, three accumulations for each sample, at a temperature of 25 °C. An accumulation consisted of 15 successive cycles [32], the final data being disclosed as mean ± standard deviation (SD).

For the average zeta (ζ) potential assessment we applied the principle of laser Doppler velocimetry technique, measuring the electrophoretic mobility of the samples, and converting it through the medium of the Helmholtz−Smoluchowsky equation:

(2) $$\zeta \;=\;\frac{4\pi \eta }{\varepsilon }\;\times \mu$$

where: μ—Electrophoretic mobility, cm^2^∙V^−1^∙s^−1^, ε—dielectric constant, nondimensional, η—solvent viscosity, Pa∙s

The measurements were performed using ultrapure water, three accumulations for each sample, at a temperature of 25 °C. An accumulation consisted of 30 successive cycles, the final data being disclosed as average 𝜁-potential ± standard deviation (SD).

For the X-ray diffraction analyses (XRD) we have used the Cu Kα characteristic radiation beam with λ = 1.5418 Å, in the 2θ = 2–60° range. Results have been collected using a Panalytical X’PERT MPD X-ray Diffractometer from Malvern Panalytical (Royston, UK).

## 3. Results

### 3.1. Fourier-Transform Infrared Spectrometry (FTIR)

**Figure 1:**
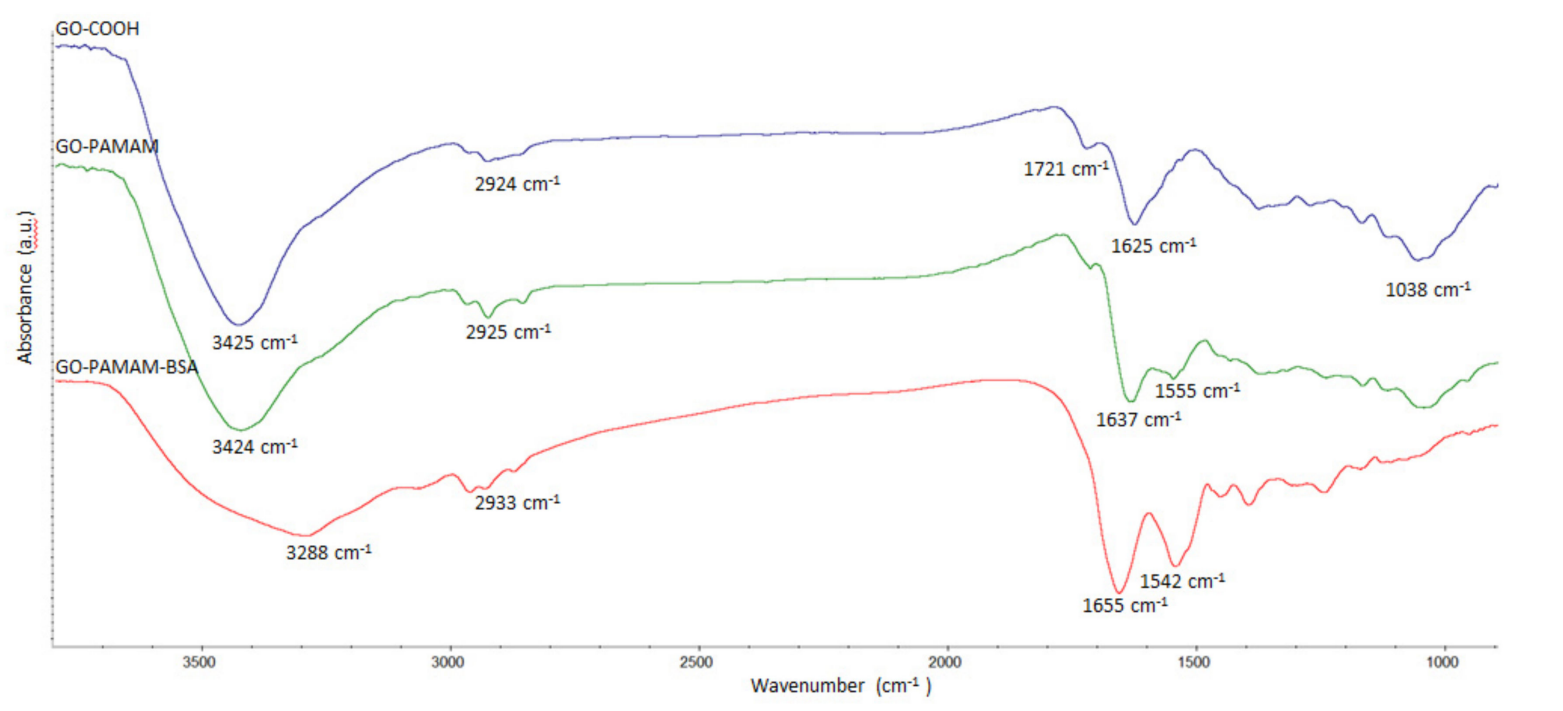
FTIR spectra of GO-COOH, GO-PAMAM and GO-PAMAM-BSA.

The FTIR spectral results of the synthesized materials are displayed in Figure 1. Signals at 1721 and 1038 cm⁻^1^ from the GO-COOH sample were attributed to the C=O and C–O, respectively [33,34] stretching vibrations of –COOH groups. The 1625 cm⁻^1^ band was attributed to COO– symmetric vibration and the 2924 cm⁻^1^ signal was given by C-H stretching vibration. The broad band at 3425 cm⁻^1^ corresponded to O-H bonds’ stretching vibrations [31]. After functionalization with PAMAM, the FTIR spectra of the newly formed complex (GO-PAMAM) revealed the covalent linkage between the GO sheets and the dendrimer. The new signal at 1555 cm⁻^1^ and the shift of the 1625 cm⁻^1^ peak to 1637 cm⁻^1^ were attributed to amidic in-plane N–H bend [35] and amidic C=O stretching vibration, respectively [36].

The large signal at 3288 cm^−1^ in GO-PAMAM-BSA was due to the high number of hydrogen bonds established between GO-PAMAM and BSA being a proof of the formation of a GO-PAMAM-BSA complex. In the case of GO-PAMAM-BSA one may notice an enlargement of the 1542 cm^−1^ peak and a shift of the 1637 cm^−1^ peak to 1655 cm^−1^ which corresponded to the formation of a bigger number of amide bonds. These changes were attributed to the complexation of GO-PAMAM with BSA.

### 3.2. X-Ray Photoelectron Spectrometry (XPS)

**Figure 2:**
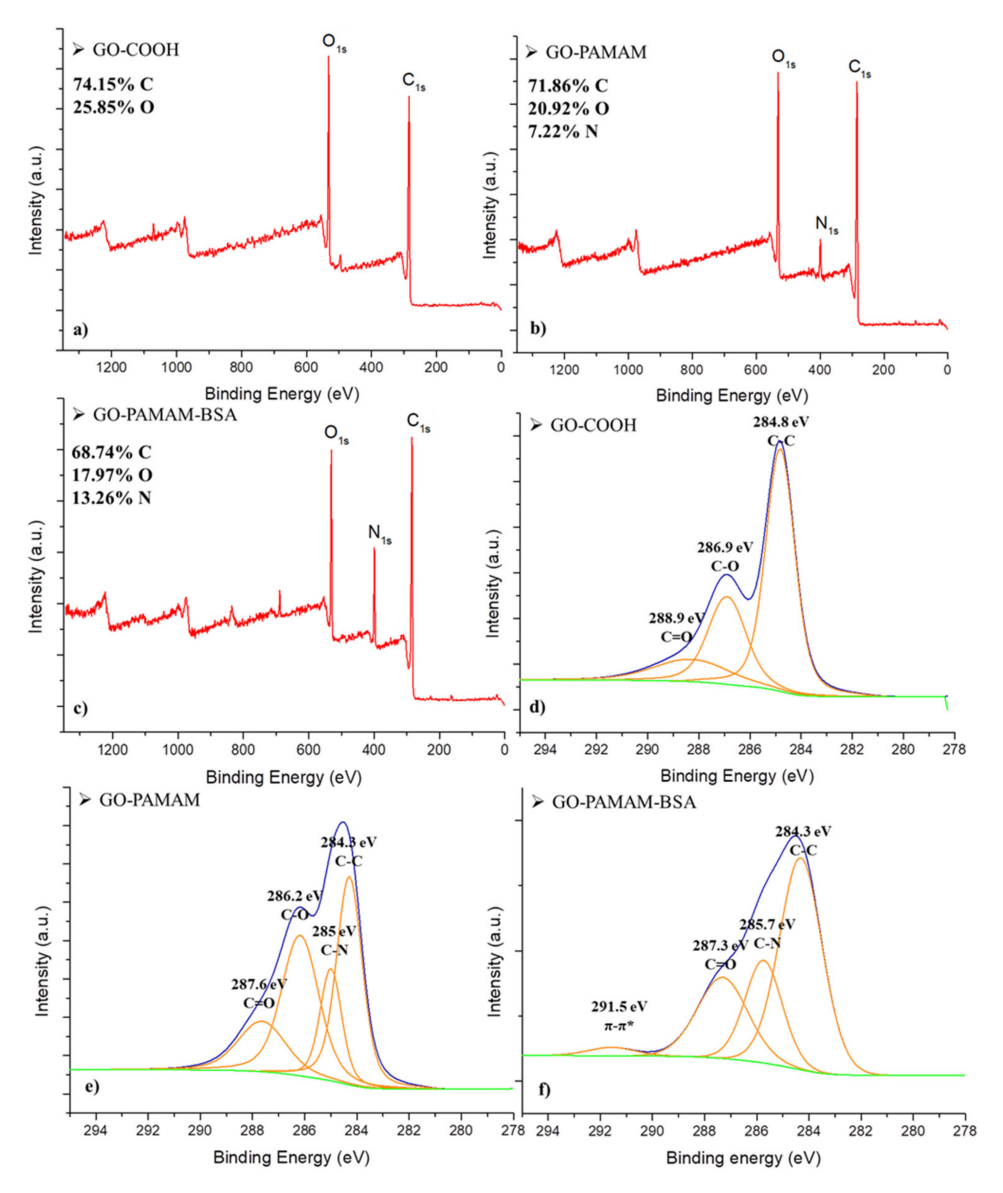
XPS (**a–c**) survey spectra and (**d–f**) C1s deconvoluted spectra of GO-COOH, GO-PAMAM and GO-PAMAM-BSA.

Figure 2a–c shows the XPS survey spectra for GO-COOH, GO-PAMAM and GO-PAMAM-BSA. The change of the atomic composition was noticed as the modification of the graphenic material occurred. The inclusion of BSA in the GO-PAMAM system made a small variation in the elemental composition. The nitrogen content increased, compared to the previous complexes and it was attributed to the presence of the protein.

Figure 2d–f represents the C1s deconvoluted spectra of the GO-COOH, GO-PAMAM and GO-PAMAM-BSA analyzed samples. Previous studies reported the appearance of C-C/C=C signal in the GO spectra at ~284.5 eV [37]. The XPS signals at ~286.8 and ~288 eV have been attributed to C-O and C=O groups from oxidized graphene samples [38]. The signal at 285 eV in GO-PAMAM and GO-PAMAM-BSA spectra was associated with a C−N bond [39] resulting from chemical bonding between GO and dendrimer, while the 291.5 eV signal was assigned to π-π* interaction expected to occur in the presence of BSA [38]. These results illustrate the successful linkage of the dendrimer and protein to the graphene surface, stated in the premise of the study.

### 3.3. Raman Spectrometry

**Figure 3:**
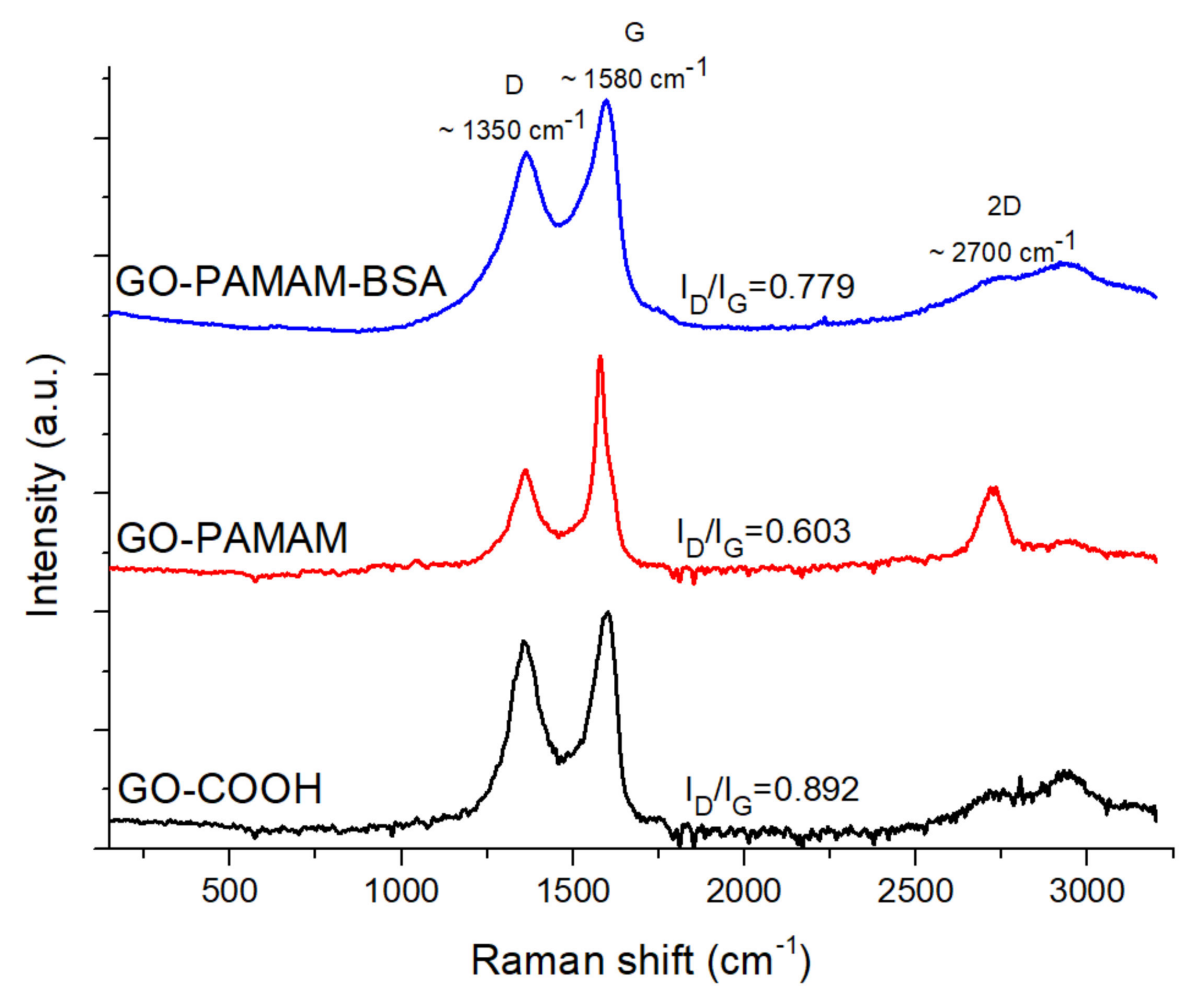
Raman spectra of GO-COOH, GO-PAMAM and GO-PAMAM-BSA.

To study the formation of the new materials, the Raman spectra for GO-COOH, GO-PAMAM and GO-PAMAM-BSA were compared (Figure 3). All the samples displayed the D and G characteristic bands at ~1350 and ~1580 cm⁻^1^. Moreover, a broad 2D band has appeared at the ~2700 cm⁻^1^ region of the GO-PAMAM-BSA Raman spectrum.

The smallest D and G bands intensities ratio (I_D_/I_G_) in the dendrimer complex indicates the existence of fewer defects [40], compared to GO-COOH or the GO-PAMAM-BSA, due to the formation of covalent and non-covalent bonds between the graphenic sheets facilitated by PAMAM chains, contributing to the decrease of the defect band (D) intensity. In addition to this, the appearance of the 2D sharper signal in the GO-PAMAM sample compared to GO-COOH, was related to the formation of structures containing a smaller number of layers [41], which was due to the penetration of PAMAM molecules between the graphenic sheets. In this case, the dendrimer presence led to the disruption of GO-COOH agglomerates. For GO-PAMAM-BSA the presence of the BSA protein led to the formation of numerous hydrogen bonds and thus re-aggregation of the GO complex.

### 3.4. X-Ray Diffraction Analysis (XRD)

X-Ray diffraction analysis is widely used for the investigation of crystallinity zones in various materials. Semicrystalline polymers can also be studied through this method, resulting in decisive information for the evaluation of surface modification processes [42].

**Figure 4:**
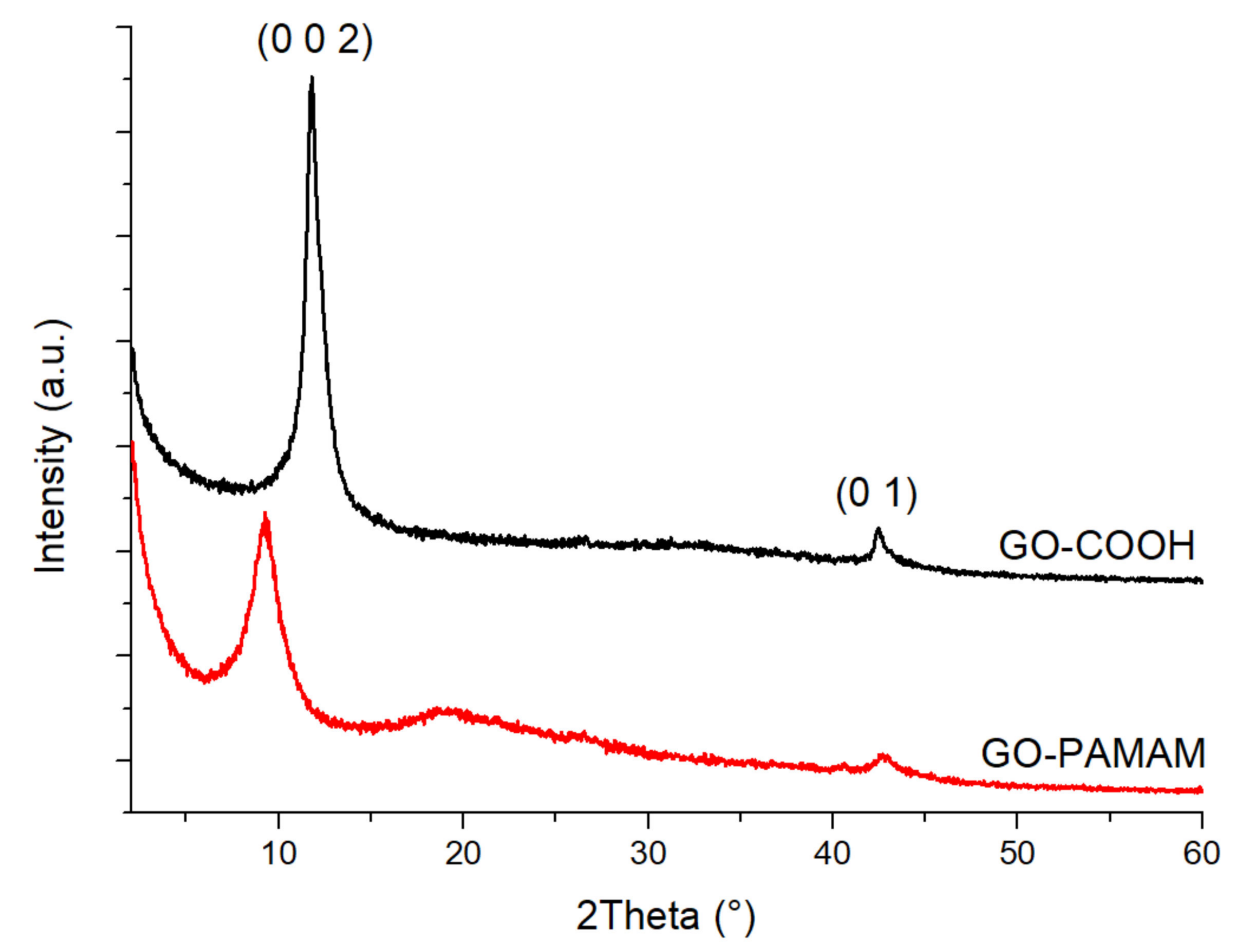
XRD results for native BSA and synthesized GO-COOH, GO-PAMAM and GO-PAMAM-BSA.

The XRD spectra (Figure 4) revealed the appearance of a diffraction peak at 2θ ≈ 9.2°, assigned to the (0 0 2) lattice [43]. It could be observed that for the GO-COOH sample the (0 0 2) diffraction plane position peak was located around 11.85°, while the one for GO-PAMAM was located around 9.24°. At the same time, the distance between the planes increased from 7.462 to 9.656 Å, which was another proof for the disintegration process of multilayered GO-COOH in the presence of PAMAM, according to Raman spectra.

### 3.5. Thermogravimetric Analysis (TGA)

**Figure 5:**
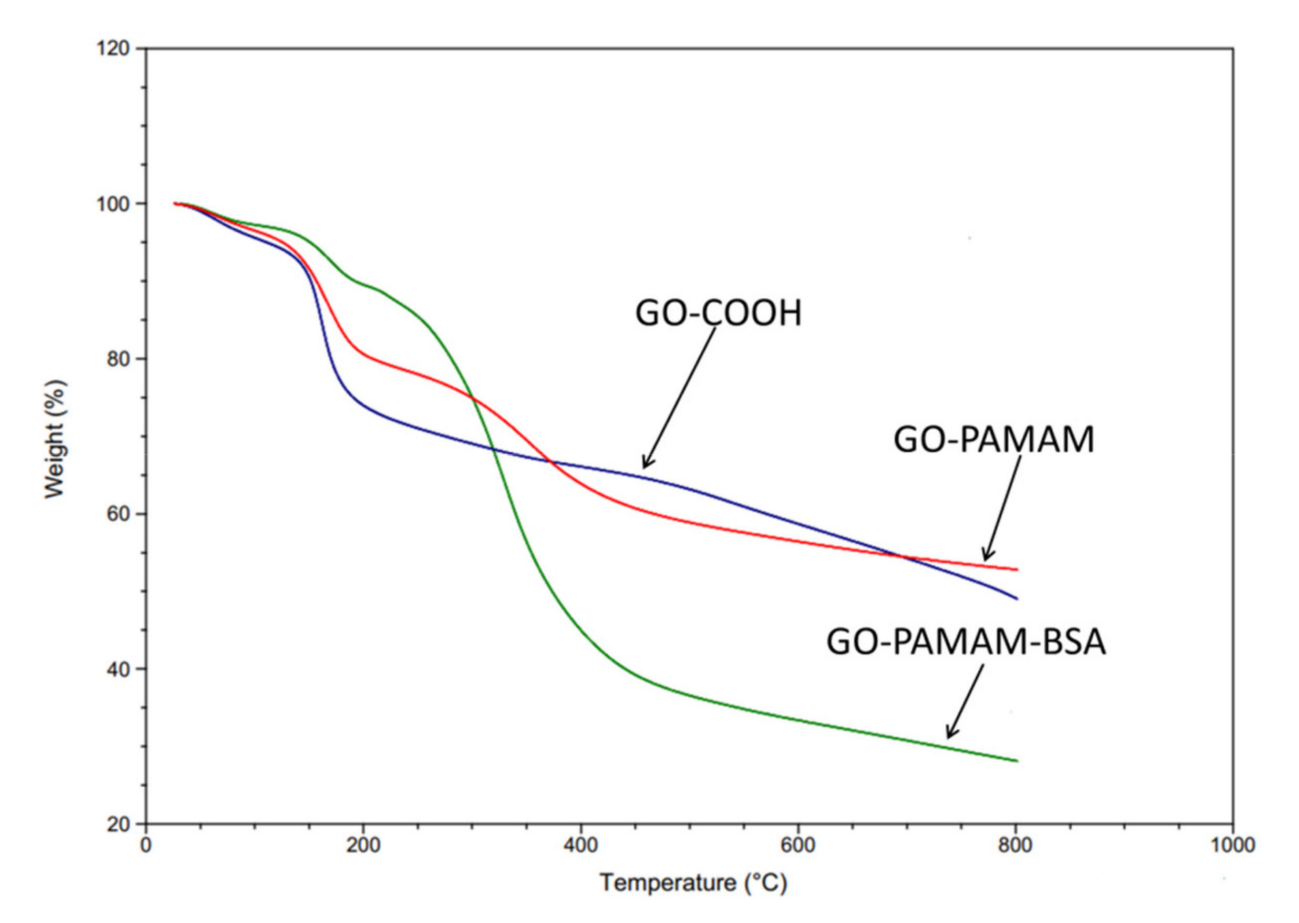
TGA curves for GO-COOH, GO-PAMAM and GO-PAMAM-BSA.

TGA analysis was used for the evaluation of the samples’ thermal stability. Figure 5 presents the thermal degradation curves for GO-COOH, GO-PAMAM and GO-PAMAM-BSA. All the samples showed a slight weight loss below 200 °C which was due to the evaporation of the remaining sample humidity. For GO-PAMAM and GO-PAMAM-BSA the appearance of a degradation step around 330 °C was observed and it corresponded to the elimination of functional groups corresponding to BSA and PAMAM dendrimer.

The protein influenced considerably the total weight loss, raising it to ~72%, a sharp mass decrease occurring at approximately 400 °C. One might notice a significant increase of the initial thermal stability for GO-PAMAM-BSA in comparison with GO-COOH and GO-PAMAM (Table 1), which was due to the multiple covalent and non-covalent bonding between the complexes formed when BSA interacted with GO-PAMAM.

### 3.6. Circular Dichroism (CD)

**Figure 6:**
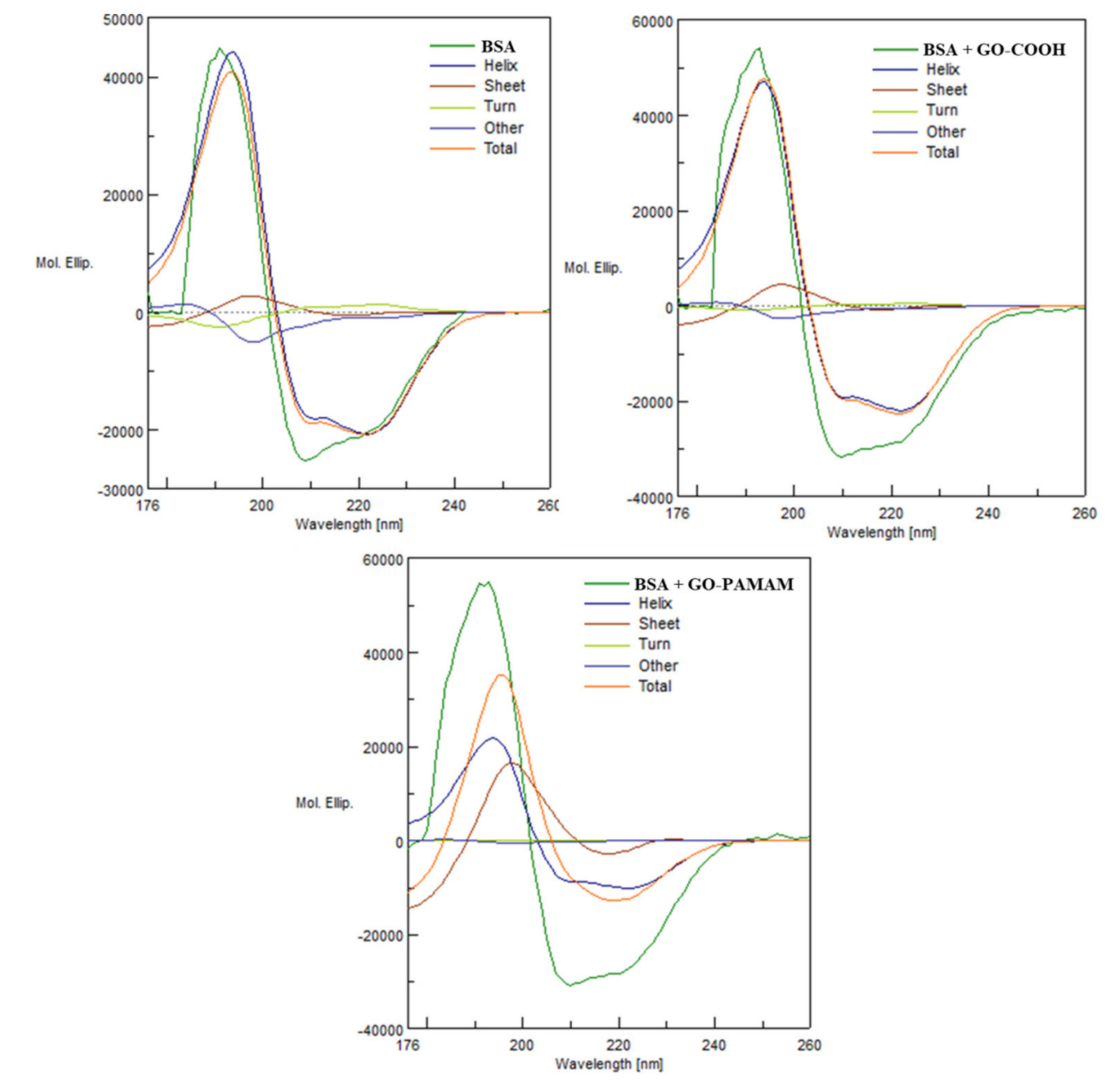
Spectral secondary structure deconvolution for circular dichroism results of BSA, GO-COOH + BSA and GO-PAMAM+BSA (GO:BSA = 1:1 wt).

In order to study the influence of the modified GO upon the protein structural integrity, CD analyses were performed for 1:1 (wt) mixture of BSA with previously synthesized GO-COOH and GO-PAMAM. The measurements were done after the components were brought in contact for 1.5 h. Spectral curves for native BSA, GO-COOH:BSA and GO-PAMAM:BSA and the results of each deconvolution into pure secondary structural components, with respect to their proportion are depicted in Figure 6. All the CD spectra exhibited a positive signal at around 192 nm, and two negative ones at 210 and 225 nm. This was the classical α-helix spectral shape obtained from circular dichroism spectrometry, denoting the significant presence of α-helix structure in the protein conformation [44].

The quantitative multivariate analysis software, including a 26 protein reference set was used in order to obtain an estimation for the protein secondary structure composition [45]. There could not be observed any important difference between the native BSA spectra and the GO-COOH:BSA mixture spectra, while the GO-PAMAM:BSA mixture spectra revealed an important modification of the protein spatial conformation. Table 2 lists the results of the secondary structure composition estimation (SSE), obtained from the collected spectra. The estimation was done via the principal component regression method (PCR). The native protein contained 67.6% α-helix. The addition of GO-COOH in a 1:1 ratio led to an increase in the α-helical content to 71.8%, after 1.5 h of interaction.

The interaction with GO-PAMAM led to a decrease in α-helical structural content to 33.2%. Therefore, the GO-PAMAM complex exhibited a significant influence against the secondary structure of BSA protein since it strongly altered the regular structure of α-helix. The β-sheet secondary structure content increased from 10.9% in BSA to 64.9% in GO-PAMAM:BSA mixture. Moreover, from Figure 6, BSA + GO-PAMAM, one could observe the shape of the spectral curve corresponding to the β-Sheet structures after the deconvolution. The positive band exhibited higher amplitude than the negative band, a characteristic for strongly twisted sheets [46]. This was strong evidence that the BSA molecules were deeply involved in various interactions with the GO-PAMAM complex.

### 3.7. Dynamic Light Scattering (DLS)

The isoelectric point of BSA has been determined to be at pH 3.1, the acidity of the protein playing an important role in forming strong interactions with the modified graphene oxide surfaces (GO-PAMAM). The amine groups form GO-PAMAM structures, alkaline in nature, provide a great connection platform between the GO surface and the biomolecule.

Table 3 contains the DLS results for GO-COOH, GO-PAMAM, and GO-PAMAM-BSA. By means of this technique the aggregate size was studied, polydispersity index and 𝜁-potential, as they are significant to a better understanding of the interaction between the components. From the size measurement results, we observed the formation of ~710 nm aggregates for the carboxylated graphene oxide, which was due to its partially hydrophobic nature. GO-PAMAM exhibited a much lower size than GO-COOH aggregates which meant that multilayer GO-COOH suffered disintegration on a large scale, leading to a few-layered GO-PAMAM structure, according with the sharp 2D band from the GO-PAMAM Raman spectrum. On the other hand, a slight size increase of the GO-PAMAM-BSA compared to GO-PAMAM was another confirmation of the protein rearrangement in the presence of the chemically modified GO, as the polydispersity index had a low standard deviation value.

In Table 3, the determined ζ-potential, as mean ± SD, is presented. The surface potential of the native protein was determined to be −17.7 ± 2.00, suggesting the stability of the protein in the aqueous solution [47,48]. The substantial electrokinetic potential variation of GO-PAMAM compared to GO-COOH was a demonstration of the successful surface modification.

## 4. Conclusions

Novel graphene oxide-based protein complex has been synthesized with potential biomedical applications. The investigation upon the compatibility of the two phases revealed an interesting affinity of the protein towards the GO-PAMAM system. Not only was the chemical association of the two components observed, but also the influence of the newly generated bonds on the secondary structure of BSA. After thorough inquiry the study showed that the expressly modified surface has a predisposition towards forming GO structures with a smaller number of layers. This is a result of the bonds that are formed between the dendrimer and the GO surface. The successful assembly of the GO-PAMAM system was also demonstrated. FTIR and XPS techniques directly indicated the bonding formation, while the DLS measurements illustrated it via the alteration in electrokinetic potential.

Furthermore, as a consequence of the modification process, the GO-PAMAM’s affinity towards protein linkage was evaluated, as well as the possible structural alterations. Spectrometric techniques along with Circular Dichroism Secondary Structure Estimation have revealed the reduction of the α-helical content with respect to the secondary structure of BSA after its interaction with the modified graphene oxide in a 1:1 ratio. The sole presence of GO-COOH in an aqueous BSA solution does not show major impact upon protein folding. In contrast, the presence of GO-PAMAM in the same conditions has tremendous impact, promoting the protein rearrangement by preferring a strongly twisted β-sheet secondary structure organization. Within the GO-PAMAM-BSA complex structure, various interactions occur between the biomolecule and graphenic phases. This provides a great start for such interaction studies, variation of BSA/GO-COOH/GO-PAMAM ratios being a matter of further studies. FTIR data showed the development of amidic bonds, while XPS results showed the existence of π-π * interactions. DLS analyses displayed changes in ζ-potential which supports that the electrostatic interaction possesses a significant role for the binding mechanism, as presence of the dendrimer promotes protein adhesion. All these mentioned interactions contribute to the assembly of a highly thermostable system. GO-PAMAM-BSA has greater thermal stability properties compared to GO-COOH or GO-PAMAM. XRD data corroborated all the previous results, being in agreement with results obtained from Raman, FTIR and XPS analyses.

The displayed data provides conclusive and innovative information referring to the new GO-PAMAM-BSA synthesized complex structure. From this study the covalent linkage between GO-COOH surface and the dendrimer was demonstrated, as well as the development of various non-covalent and covalent interactions between the expressly modified GO-PAMAM and BSA protein. The structural alterations of the protein have also been discovered, the new system exhibiting improved properties. On account of these results we can presume that the GO-PAMAM-BSA material has great potential regarding biomedical applications.

## Figures and Tables

**Table 1:** Weight loss percentage and weight loss temperatures for synthesized GO-COOH, GO-PAMAM and GO-PAMAM-BSA.

	Weight Loss (%) [25–800 °C]	Weight Loss Temperature (°C)		
		3%	5%	10%
GO-COOH	51.0	76	111	151
GO-PAMAM	47.2	89	125	157
GO-PAMAM-BSA	71.9	111	151	192

**Table 2:** Secondary structure composition of the protein in native form, and after 1.5 h interaction with GO-COOH and GO-PAMAM (GO:BSA = 1:1 wt).

	Secondary Structure Composition (%)			
	α-Helix	β-Sheet	Turn	Other
BSA	67.6	10.9	5.6	15.9
GO-COOH + BSA	71.8	17.6	2.2	8.4
GO-PAMAM + BSA	33.2	64.9	0.0	1.9

**Table 3:** Particle size, PdI and 𝜁-potential of native BSA, GO-COOH, GO-PAMAM and GO-PAMAM-BSA as obtained by DLS measurements.

	Size(nm) ± SD	PdI ± SD	𝜁-potential ± SD
GO-COOH	710.2 ± 20.56	0.585 ± 0.039	−32.6 ± 0.473
GO-PAMAM	293.7 ± 2.25	0.314 ± 0.012	−22.5 ± 1.19
GO-PAMAM-BSA	304.4 ± 8.145	0.382 ± 0.039	−18.6 ± 0.814
